# Correction: Mancini et al. A Novel Strategy for the Assessment of Radon Risk Based on Indicators. *Int. J. Environ. Res. Public Health* 2021, *18*, 8089

**DOI:** 10.3390/ijerph192315901

**Published:** 2022-11-29

**Authors:** Simona Mancini, Martins Vilnitis, Michele Guida

**Affiliations:** 1 Department of Computer Engineering, Electrical Engineering and Applied Mathematics (DIEM), Laboratory “Ambients and Radiations (Amb.Ra.)”, University of Salerno, 84084 Fisciano, Italy; 2 Institute of Construction Technology, Faculty of Civil Engineering, Riga Technical University, 1658 Riga, Latvia

## Figure Legend

In the original publication [1], there was a mistake in the legend for Figure 3. Diagram of the sources (brown square boxes), processes (green round boxes), and parameters that a global dynamic radon model must consider. The time-dependent parameters are in blue [20]. The original copyright owner for this figure picture should be Doctor Lluis Font Guiteras. The correct legend appears below. The authors apologize for any inconvenience caused and state that the scientific conclusions are unaffected. The original publication has also been updated.

**The correct Legend**

Figure 3. Diagram of the sources (brown square boxes), processes (green round boxes), and parameters that a global dynamic radon model must consider. The time-dependent parameters are in blue. Figure by Font L, available at https://icnts2008.bo.infn.it (accessed on 15 December 2021). Reprinted with permission from Lluis Font (2008).
